# Multilevel Factors and Sleep in Adults With Inflammatory Bowel Disease: A Qualitative Study

**DOI:** 10.1093/crocol/otae075

**Published:** 2024-12-07

**Authors:** Samantha Winders, Linda Yoo, Margaret Heitkemper, Kendra Kamp

**Affiliations:** Department of Biobehavioral Nursing and Health Informatics, University of Washington School of Nursing, Seattle, WA, USA; Department of Biobehavioral Nursing and Health Informatics, University of Washington School of Nursing, Seattle, WA, USA; Department of Biobehavioral Nursing and Health Informatics, University of Washington School of Nursing, Seattle, WA, USA; Department of Biobehavioral Nursing and Health Informatics, University of Washington School of Nursing, Seattle, WA, USA

**Keywords:** sleep, inflammatory bowel disease, health, social factors, social determinants of health

## Abstract

**Background:**

This study aimed to describe the patient-reported factors that impact sleep among individuals with inflammatory bowel disease (IBD), aligning with the Social Ecological Model of Sleep. This addresses the gap in IBD sleep research, which predominantly focuses on individual-level factors and their impact on sleep.

**Methods:**

Adults (ages 18-65) with IBD were recruited online through ResearchMatch in June 2023. Participants filled out survey questions on their demographic characteristics, health history, sleep, and IBD-related symptoms. Content analysis was conducted on 2 open-ended questions about factors that impacted their sleep.

**Results:**

This analysis included 163 adults with IBD (*M* = 39 years of age, 76.7% White, 91.4% non-Hispanic or Latino, 66.9% female, and 83.4% active IBD) who answered open-ended questions with comments about their sleep. Most participants indicated an individual-level factor impacted their sleep quality (85.3%, *n* = 139), categorized into 5 subthemes: Mental health, health, behavior and choices, physiology, and attitudes. Additionally, participants (43.6%, *n* = 71) mentioned social-level factors divided into 7 subthemes: Family, work, home, neighborhood, social network, and school. A smaller group of participants (17.2%, *n* = 28) mentioned societal-level factors designated into 4 subthemes: Natural environment and geography, technology, 24/7 society, and economics.

**Conclusions:**

This study highlights the need for tailored sleep interventions for those with IBD that consider not only disease activity but also mental health, family, work, and the natural environment. IBD clinics should prioritize sleep health using an interdisciplinary approach to holistically address the unique needs of those with IBD.

## Graphical Abstract

**Graphical Abstract:**
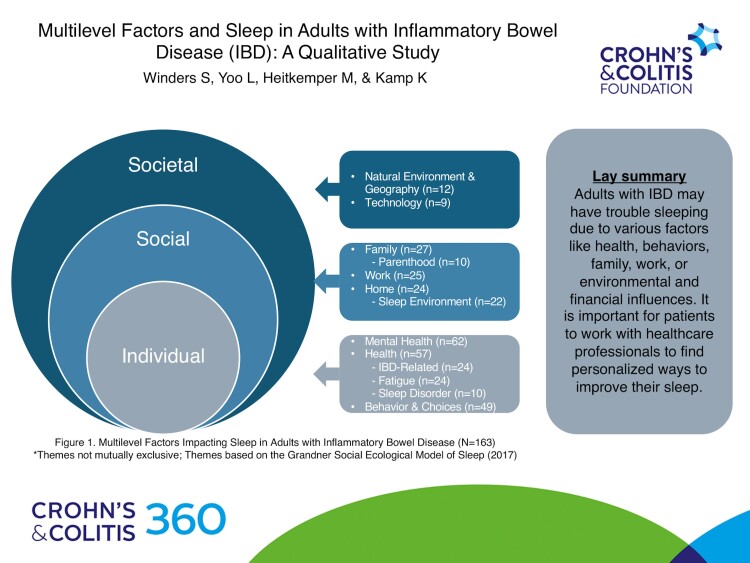

## Introduction

Sleep health, essential for physical and mental well-being, is an important concept for individuals with inflammatory bowel disease (IBD), an immune-mediated disease.^1,2^ IBD is an umbrella term for Crohn’s disease (CD) or ulcerative colitis (UC) diagnoses, affecting approximately 2.4 million people in the United States.^3^ Sleep health is closely associated with immune function.^4^ In the context of IBD, poor sleep may lead to increased rates of disease activity, hospitalization, surgery, as well as delayed healing.^4^ Both those with active and inactive IBD report poor sleep quality and high rates of fatigue.^5^ Even though 70% of patients are interested in discussing sleep as part of their IBD care,^6^ sleep is an underrecognized issue within most IBD clinics.

Apart from the commonly reported poor sleep quality in this population, social determinants of health (SDoH) may further contribute to poor sleep quality. Social determinants of health are conditions within the environment where one is born, lives, works, worships, plays, and ages that may affect health risks and outcomes.^7^ Evidence shows individuals from historically underserved communities in the United States are disproportionately impacted, with race, socioeconomic status, employment, and neighborhood being linked to sleep outcomes.^8,9^ The Social Ecological Model of Sleep includes SDoH and outlines factors across 3 levels influencing sleep: Individual, social, and societal.^10^ Individual-level factors such as sleep beliefs, attitudes, behaviors, and IBD disease activity directly influence sleep within a person. There are also social and societal-level factors (eg, sleep environment, neighborhood, natural environment) that impact sleep.

A recent systematic review identified the majority of published studies focus on the individual-level factors that influence sleep, such as age and disease activity, while only a few have measured SDoH.^11^ Prespecified answer choices may fall short of capturing these multilevel factors; thus, qualitative research provides 1 avenue to further understand the factors that influence patients’ sleep. However, few studies have examined sleep and IBD through a qualitative lens. The few who do, have reported participants do recognize the link between sleep, IBD, and mental health and want sleep interventions tailored to their needs.^6,12^ Thus, there is a need to elucidate the multilevel factors shaping sleep health among individuals with IBD. Specifically, we aim to describe the patient-reported factors that impact sleep among individuals with IBD. Results will inform future sleep interventions aimed at addressing the multilevel factors that influence sleep among people with IBD, ultimately working toward promoting sleep health equity.

## Materials and Methods

### Participants

This qualitative study recruited participants in June 2023 from ResearchMatch, a nonprofit program funded by the National Institutes of Health, as a part of the Clinical Translational Science Award program.^13^ The target population was individuals with IBD (either CD or UC) aged 18-65 years who could read and write in English.

### Procedures

Adults with IBD (aged 18-65 years), identified via ResearchMatch, were sent an email link to the survey. Potential participants were provided an overview of the study and instructed to continue the survey, indicating their consent. Next, participants completed screening questionnaires, and those eligible continued the survey. The survey contained a variety of open-ended, sleep, and demographic questions. Participants were given the option to skip or provide no information on any questions. At the end of the study, participants could provide their email if they wanted to receive a $15 gift card.

### Variables and Measures

#### Demographics and disease activity

Demographic information included age, gender, biological sex, race, ethnicity, the highest level of education, employment status, and marital/partner status. Disease activity was measured using the Harvey–Bradshaw Index (HBI)^14^ for CD and the Simple Clinical Colitis Activity Index (SCCAI)^15^ for UC. The HBI asks about general well-being, abdominal pain and masses, liquid or soft stools, and extracolonic complications; a score of 5 or greater indicates active disease.^16^ The SCCAI asks about their number of bowel movements during the day and night, urgency, blood in the stool, general well-being, and extracolonic complications; a score of 3 or greater indicates active disease.^17^

#### Sleep

Sleep disturbance was measured using the Patient-Reported Outcomes Measurement Information System (PROMIS) short-form measures. This survey included 4 questions related to sleep over the past week. Participants respond on a 5-point Likert scale from “never” to “always.” PROMIS measures are valid and reliable. The average score for these measures is 50 for the US general population, while a higher score indicates more severe symptoms.^18^

#### Factors impacting sleep

Open-ended questions about factors that may interfere with sleep included “Any comments about this week’s sleep?” and “Did anything happen that could have affected this week’s sleep?.” A recall period of 1 week was selected to align with the recall period for the PROMIS sleep disturbance measure.

### Data Analysis

Data were collected and stored in Research Electronic Data Capture (REDCap).^19,20^ Categorical variables are reported using numbers and frequencies, and numerical variables are reported using means and standard deviations (SDs). For the open-ended questions, content analysis was conducted.^21^ Two researchers (S.W. and L.Y.) reviewed and coded the open-ended text using the factors in Grandner’s Social Ecological Model of Sleep.^10^ Open-ended answers were coded separately and reviewed by a second researcher. Disagreements were discussed with a third researcher (K.K.). Themes are reported as numbers representing the number of participants who mentioned each theme. Themes with less than 5% of responses were removed. Independent-samples proportion tests were run to test differences in responses between the proportion of participants among diagnosis (CD or UC) and disease activity (active or inactive) subgroups. All data analyses were conducted in SPSS (version 29.0.0.1).

### Ethical Considerations

Institutional Review Board approval was obtained, and all participants gave informed consent.

## Results

Out of 250 participants who completed the online survey, 163 filled out the open-ended questions with substantive comments about their sleep. The 87 responses excluded from the 250 participants, were responses as “no,” not enough information (eg, “no additional comments,” “no significant events,” and “NA”), or no response. There were statistically significant differences in race (*P* = .03) and marital status (*P* = .01) between those who provided comments about sleep and those who did not.

### Demographics and Disease Activity

Those who filled out the open-ended questions with comments about their sleep were, on average, 39 (SD 11.0) years of age (range: 19-65), 76.7% (*n* = 125) white, 91.4% (*n* = 149) non-Hispanic or Latino, and 66.9% (*n* = 109) female. Additionally, 54.6% (*n* = 89) had UC and 83.4% (*n* = 136) had active IBD (Table 1). PROMIS scores for sleep disturbance ranged from 32 to 73 (*M* = 56.7, SD = 8.1).

**Table 1.** Participant characteristics for individuals with inflammatory bowel disease that responded with comments about sleep (*N* = 163).

	Participants (*N* = 163)
IBD diagnosis, *n* (%)	
Crohn’s disease	74 (45.4)
Ulcerative colitis	89 (54.6)
Age, *n*	153
Mean (SD)	39 (11.0)
Range	19-65
Race, *n* (%)	
White	125 (76.7)
Black	30 (18.4)
Other	6 (3.7)
No answer	2 (1.2)
Ethnicity, *n* (%)	
Hispanic or Latino	10 (6.1)
Non-Hispanic or Latino	149 (91.4)
No answer	4 (2.5)
Biological Sex, *n* (%)	
Female	109 (66.9)
Male	53 (32.5)
No answer	1 (0.6)
Disease activity, *n* (%)	
Active	136 (83.4)
Inactive	27 (16.6)

Abbreviations: IBD, inflammatory bowel disease; SD, standard deviation.

### Multilevel Factors

There were 3 main themes based on Grandner’s Social Ecological Model of Sleep (individual-, social-, and societal-level factors). Additionally, 15 of the 27 subthemes from the model were identified. Subgroup analyses for UC compared to CD and active disease compared to inactive disease are presented in Table 2. Illustrative quotes for themes are presented in Table 3.

**Table 2.** Multilevel factors impacting sleep in adults with inflammatory bowel disease by diagnosis and disease activity.

Theme	Total (*N* = 163)	Active disease (*n* = 136)	Inactive disease (*n* = 27)	*P*-value	CI	Crohn’s disease (*n* = 74)	Ulcerative colitis (*n* = 89)	*P*-value	CI
**Individual factors**									
Mental health	62	48	14	.05	(−0.04 to 0.36)	30	32	.27	(−0.10 to 0.19)
Health	57	47	10	.40	(−0.16 to 0.23)	32	25	.02^a^	(0.00-0.29)
IBD-related	24	23	1	.04^a^	(−0.22 to 0.01)	16	8	.01^a^	(0.01-0.24)
Fatigue	24	20	4	.50	(−0.13 to 0.17)	14	10	.08	(−0.04 to 0.19)
Sleep disorder	10	8	2	.38	(−0.08 to 0.16)	8	2	.01^a^	(0.00-0.17)
Behavior and choices	49	41	8	.48	(−0.18 to 0.19)	23	26	.40	(−0.12 to 0.16)
**Social factors**									
Family	27	19	8	.02^a^	(−0.01 to 0.34)	12	15	.46	(−0.12 to 0.11)
Parenthood	10	7	3	.12	(−0.05 to 0.21)	6	4	.17	(−0.04 to 0.12)
Work	25	21	4	.47	(−0.14 to 0.16)	9	16	.15	(−0.17 to 0.06)
Home	24	23	1	.04^a^	(−0.22 to 0.01)	8	16	.10	(−0.18 to 0.04)
Sleep environment	22	21	1	.05	(−0.20 to 0.02)	7	15	.08	(−0.18 to 0.03)
**Societal factors**									
Natural environment and geography	12	8	4	.05	(−0.04 to 0.25)	4	8	.19	(−0.12 to 0.05)
Technology	9	7	2	.32	(−0.07 to 0.16)	4	5	.48	(−0.08 to 0.08)

Physiology, attitudes, neighborhood, social network, school, 24/7 society, and economics themes were removed due to 5% cut off. Themes are not mutually exclusive.

^a^ *P* < .05.

**Table 3.** Illustrative quotes from individuals with inflammatory bowel disease regarding factors which affected this week’s sleep (*N* = 163)

Theme (*N*)^a^	Description	Illustrative quote
**Individual factors**		
Mental health (*n* = 62)	Included statements related to mental well-being, such as anxiety or stress.	“Anxiety, lots of pressure from life, poor sleep schedule, waking up multiple times throughout night.” *[UC, age 37, female]*
Health (*n* = 57)	Included statements related to sleep being impacted by illness, pain, sleep disorders, or another medical condition (such as IBD) that interfered with sleep.	“Recovering from a URI has made me feel like I need more sleep and I have less control over that need.” *[CD, age 27, female]*
IBD-related (*n* = 24)	Included statements about GI-related symptoms as well as extraintestinal manifestations.	“For 20+ years, abdominal pain, back pain, joint pain, and crohn’s issues keep waking me up.” *[CD, age 65, female]*
Fatigue (*n* = 24)	Included statements regarding symptoms of fatigue and tiredness indicating general fatigue or nonspecific fatigue.	“I slept lesser [sic] than I did last week because I always feel tired before sleeping who h [sic] impacts my sleep.” *[CD, age 36, male]*
Sleep disorder (*n* = 10)	Included statements which mentioned a sleep disorder.	“I currently take trazadone for my chronic insomnia, it worked well at first but isn’t working well anymore. I have a hard time gaming [sic] sleep, wake up a couple times overnight, and don’t feel particularly rested.” *[CD, age 41, female]*
Behavior and choices (*n* = 49)	Included statements related to personal actions that impacted sleep health, such as alcohol use, herbal supplements, changes or irregular sleep schedules, travel (leisure or work), and napping.	“My sleeping experience have [sic] been poor because I sleep late and use alcohol to support my sleep which affect my ability to wake up early.” *[UC, age 35, male]*
Physiology (*n* = 6)	Included statements related to the circadian rhythm and dreaming.	“Hormones at ovulation and PMS affect my sleep. Cramps affect my sleep. ADHD and late circadian rhythm affects my sleep.” *[CD, age 42, female]*
Attitudes (*n* = 4)	Included statements related to confidence about sleep, control of sleep, and positive attitudes and beliefs toward sleep.	“I’ve been sleeping well. I feel good about how I’ve been able to keep a consistent sleep schedule.” *[UC, age 29, female]*
**Social factors**		
Family (n = 27)	Included statements related to interruptions to sleep due to nuclear or extended family relations.	“As with most weeks, I have a lot going on as far as taking care of my elderly mother, helping my husband with appointments and doctors visits for his health related needs as well as trying to take care of my own wellness. I feel exhausted at the end of the day but somehow still end up tossing and turning during the night.” *[UC, age 55, female]*
Parenthood (*n* = 10)	Included statements related to parenting a child.	“I have a 6 month old that I still breastfeed during the night.” *[CD, age 38, female]*
Work (*n* = 25)	Included statements related to the demands or stress from work.	“I couldn’t sleep well this week because I’ve been concern [sic] about work stress.” *[CD, age 25, male]*
Home (*n* = 24)	Included statements related to the individual’s most proximal environment which included their sleep environment, social dynamics, and other related stressors within the home.	“Avoiding my roommate makes me lay in bed earlier but might also lead to more phone time and falling asleep later.” *[UC, age 23, female]*
Sleep environment (*n* = 22)	Included statements specific to the sleep environment such as pertaining to the bed, pillows, light, noise, temperature, etc.	“I’ve been under a lot of stress. Additionally, I’m currently sleeping on a family member’s sofa. The pillows have been uncomfortable but more so the sun coming in through the living room window, even with extra curtains, wakes me up every morning regardless of if I’m ready to wake up or when I went to sleep.” *[UC, age 32, female]*
Neighborhood (*n* = 7)	Included statements related to the neighborhood including light, noise, crime, and stress as a product of the built environment.	“Construction across the street (I work nights, so then working in the early morning hours when I’m trying to sleep doesn’t help me stay asleep).” *[UC, age 34, male]*
Social network (*n* = 5)	Included statements related to an individual’s relations with friends and acquaintances outside of the immediate family.	“I spent two night’s [sic] sleeping at a friend’s house.” *[UC, age 42, female]*
School (*n* = 2)	Included statements related to the demands or stress from school.	“I have clinicals for nursing school Thursdays and Fridays, so I only get about 6 hours of sleep on Wednesday and Thursday. I attended a birthday party late at night in Saturday and had to wake up early on Sunday. I also have an exam tomorrow I’ve been staying up late to study for.” *[CD, age 26, female]*
**Societal factors**		
Natural environment and geography (*n* = 12)	Included statements related to the physical environment and geography such as weather and sunlight.	“Humidity, hot flashes, heat, menopause, full moon disrupt sleep” *[UC, age 55, female]*
Technology (*n* = 9)	Included statements related to using technology in the bedroom such as the use of internet, phones, or television.	“I haven’t been sleeping very well this week, it’s mostly because I [sic] been spending too much time on the internet before going to bed. It reduces my sleep time because I have to be up the next morning.” *[UC, age 26, female]*
24/7 Society (*n* = 5)	Included statements related to the increased demands of modern society to do things around the clock, including shift work.	“I work overnight 3 nights a week so my sleep schedule flip flops.” *[CD, age 40, female]*
Economics (*n* = 2)	Included statements related to financial demands or stresses.	“Pressure from work and bills” *[UC, male]*

Abbreviations: UC = ulcerative colitis, CD = Crohn’s disease.

^a^Not mutually exclusive.

### Individual-Level Factors

Most participants stated an individual-level factor impacted their sleep health (*n* = 139). Five of Grandner’s 7 subthemes were mentioned by the participants: Mental health, health, behavior and choice, physiology, and attitudes. The most common subtheme was mental health (*n* = 62), which included statements related to mental well-being, such as anxiety, impacting sleep health. Participants described “anxiety, lots of pressure from life,” “high levels of stress,” “intrusive thoughts and racing mind,” and “constant thinking” as impacting sleep health. The second most common subtheme was health (*n* = 57), with 3 categories of health: IBD-related, sleep disorders, and fatigue. IBD-related health that impacted sleep health included gastrointestinal symptoms and IBD flares, effects of medications, and nocturnal bowel movements. Participants described IBD-related health as “abdominal pain,” “bowel irregularities,” and “empty my ostomy bag.” The sleep disorders category included “insomnia,” “restless legs syndrome,” and devices used for sleep disorders such as “bipap machine.” Fatigue was described by participants as “exhausted and run down constantly,” “sleepy days,” “completely drained and exhausted,” “feeling tired,” and “drowsy by midday.” The behavior and choice subtheme (*n* = 49) included actions that impacted sleep health, such as alcohol use, herbal supplements, changes or irregular sleep schedules, travel (leisure or work), and napping. The physiology and attitudes subthemes had less than 5% of the total responses. More participants with CD stated the themes/subthemes of health (*P* = .02; CI = 0.00-0.29), IBD-related (*P* =.01; CI = 0.01-0.24), and sleep disorder (*P* = .01; CI = 0.00-0.17) impacted their sleep health. Additionally, more participants with active disease stated the IBD-related subtheme (*P* = .04); CI = −0.22 to 0.01) impacted their sleep health.

### Social-Level Factors

Several social-level factors were identified by participants to impact their sleep (*n* = 71). Six of Grandner’s 10 subthemes were mentioned by participants with IBD: Family, work, home, neighborhood, social network, and school. The most common subtheme was family (*n* = 27), with a subcategory of parenthood (*n* = 10). Participants made statements about interruptions to sleep due to nuclear or extended family relations stating, “breastfeeding during the night,” “family stress,” “taking care of my elderly mother,” and “arguing with my partner.” The second most common subtheme was work (*n* = 25) and included statements by participants about demands or stress from work, such as “work stress,” “heavy workload,” and “pressure from work.” The third most common subtheme was home (*n* = 24) with a subcategory of sleep environment (*n* = 22), which was composed of statements relating to “avoiding my roommate,” “pillows have been uncomfortable,” “sun coming in through the living room window,” and “the room is so warm that I can’t use a blanket.” The neighborhood, social network, and school themes had less than 5% of total responses. There were no differences between the CD and UC groups. However, more participants with active disease stated family (*P* = .02; CI = −0.01 to 0.34) and home (*P* = .04; CI = −0.22 to 0.01) impacted their sleep health.

### Societal-Level Factors

Societal-level factors impacting sleep health were mentioned by 28 of the 163 participants. Four of Grandner’s 7 subthemes were mentioned by participants: natural environment and geography, technology, 24/7 society, and economics. The natural environment and geography subtheme (*n* = 12) included factors related to weather and the outside environment, such as “humidity,” “full moonlight,” “storm 2 nights ago,” and “wind howling and blowing limbs.” The technology subtheme (*n* = 9) included factors related to phone use and “spending too much time on the internet.” The 24/7 society and economics subtheme had less than 5% of total responses. There were no statistically significant differences between diagnosis (CD or UC) or disease activity (active or inactive) subgroups.

## Discussion

In their responses, individuals with IBD reported a variety of factors that impacted their sleep over the past week. These factors were categorized to align with Grandner’s Social Ecological Model of Sleep. The top 3 individual-level factors mentioned were related to mental health, health, and behavior and choices. Common social-level factors included family, work, and sleep environment, while societal-level factors included the natural environment and technology.

The most common individual-level factor was related to anxiety, stress, and worry impacting sleep (mental health). Life stressors can interrupt sleep and result in poor sleep, which may affect the ability to cope with these stressors. Previous evidence has found that daily stressors may lead to a 13% increase in the risk of insomnia for every additional stressor in adults.^22^ However, this could depend on an individual’s sleep reactivity, which is one’s tolerance to stress-related sleep disturbance.^23^ Determinants of sleep reactivity include gender, cognitive emotional factors, and the type of stressor.^23^ Health outcomes and sleep share a similar bidirectional relationship. Good quality sleep is beneficial to physical, mental, and emotional health. Participants reported several health concerns that impacted their sleep, including IBD symptoms, sleep disorders, and fatigue. Sleep problems, including sleep disorders, are common in those with IBD and result in a greater risk for inflammation, relapse, and poor outcomes.^5^ Notably, the health theme and subthemes of IBD-related and sleep disorder responses varied between CD and UC groups with more participants with CD in each theme/subtheme. The IBD-related subtheme also varied between active and inactive disease groups with more participants with active disease as expected. Future interventions should focus on improving sleep in those with IBD through adapting coping strategies for daily stressors, especially during flares. Future research should further explore the impact of diagnosis type on sleep.

Family was the most prevalent social-level factor mentioned by those in our survey. Stressors related to nuclear or extended family relations may lead to disruptions in an individual’s sleep. Previous research has found family contact, strain, and support were predictors of troubled sleep among adults.^24^ Individuals with IBD are often diagnosed in young adulthood (peak onset of 15-29 years old),^25^ which could be a time of starting to live independently, leaving home for college, and starting their own family. This could result in changing family relations and added responsibilities. The family and home theme responses varied between disease activity groups with a greater proportion of participants with active disease stating both themes. This may highlight a greater need for intervention pertaining to family and home factors impacting sleep in those with active disease. In addition, one’s sleep environment plays a major role in the quality of sleep one gets. Participants reported discomfort in their bed, pillows, temperature, and amount of light. The thermal environment, impacted by air conditioners and outside temperatures, may lead to one’s body temperature being too warm or cold. The body’s core temperature can lead to a disruption in sleep; for example, warm temperatures can reduce the length of rapid-eye movement cycles and increase wakefulness periods. Light exposure has also been known to disrupt sleep, resulting in the misalignment between the external environment and the internal circadian clock.^26^ The stress and demands of work and shift work were also mentioned to have impacted these individuals’ sleep. These daily demands of work impacting sleep may then lead to decreased work productivity and interpersonal conflicts. Previous implementation of workplace interventions focused on improving employee health has shown significant improvements in sleep quality, work stress, anxiety, and depression.^27^

The natural environment and geography led to disruptions in the sleep of participants. Humidity, heat, storms, and full moonlight were all reported. As previously mentioned, temperature and light may lead to disruptions in body temperature and the circadian clock. Climate change has led to increased nightly temperatures across the United States, which is predicted to increase the number of nights of insufficient sleep.^28^ Although most of these natural environment factors are outside of the individual’s control, room darkening curtains or indoor temperature controls may mitigate these factors’ impact on sleep. Technology was also commonly mentioned by participants in our study. Technology use at bedtime leads to blue light exposure, suppressing melatonin release, and can lead to irregular sleep schedules and bedtime procrastination.^29^ In order to promote good sleep habits, the Centers for Disease Control and Prevention (CDC) recommends maintaining a consistent sleep schedule which includes removing electronic devices from the bedroom.^30^ On the other hand, a few participants noted the use of technology to help them sleep, which highlights the need for individualized interventions.

Few studies have taken a qualitative approach to examining factors that influence sleep in IBD. Thus, this study addresses a critical gap by incorporating patient voices to understand the individual, social, and social factors that may influence sleep among individuals with IBD. Previous IBD studies have primarily focused on individual-level factors, specifically age and disease activity.^11^ Our study adds to the literature by further highlighting the importance of considering mental health, the impact of family and work, and the natural environment, which all work together to influence sleep health. Although participants did not mention all subthemes in Grandner’s model (genetics, beliefs, religion, culture, race/ethnicity, socioeconomics, globalization, public policy, racism, and discrimination), these factors still may be contributing to sleep disturbance. However, we found participants mentioned subcategories of health (IBD-related, sleep disorders, fatigue), home (sleep environment), and family (parenthood) that were not captured in the current Social Ecological Model of Sleep,^10^ which could be unique to this population and should be considered in future adaptations of this model. Additional qualitative research is needed to further examine these subthemes and their impact on sleep. Future sleep interventions should be tailored to individual needs, considering these multilevel factors and their potential impact on intervention engagement, such as caregiving responsibilities. Future IBD research should explore the impact these SDoH may have on sleep and advocate for policy changes to address these upstream factors.

### Clinical Implications

Sleep is a critical element of health for those with IBD that clinicians must address. It has been found that patients want IBD providers to ask and give recommendations on sleep.^6^ Thus, clinicians should assess patients’ sleep and make necessary referrals. Although IBD symptoms can impact sleep health, other factors, such as mental health, could be the main driver for poor sleep in individuals with IBD. Healthcare professionals should screen for anxiety and depression during clinic visits. Cognitive Behavioral Therapy for Insomnia (CBT-I), which works to change thought patterns and behaviors that negatively impact sleep health, has been found to be feasible and acceptable by those with IBD.^31^ This might be a promising treatment for those with IBD who have insomnia. Additionally, an interdisciplinary team to address sleep issues is needed, as it is evident that cognitive issues and SDoH can influence sleep health. Social workers, therapists, and nurses can help mitigate the negative impact of social and societal-level factors that impact the lives of those with IBD as well as their sleep health.

### Limitations

This study recruited an online sample of adults with IBD across the United States. However, only the IBD diagnoses of CD and UC were included, not indeterminate colitis. Individuals with pre-existing sleep challenges may have been more likely to participate in the study. Additionally, most of our sample were women and well educated, so this may impact the responses. The online nature of the study means that we relied on patient self-report and do not have access to patient medical records for more objective disease activity measures or verification of self-reported sleep disorders. Similar to other qualitative sleep IBD studies, we relied on qualitative data obtained via written surveys. Thus, we were unable to probe with follow-up questions as interviews or focus groups allow. Additionally, the written survey answers might have led to fewer responses on certain subthemes if the participant felt as if their experience was already well captured by the survey items. Further, these written surveys only asked about sleep within the past week, which may not be representative of a participant’s overall sleep. However, this time frame was selected to align with the recall period of the PROMIS measures.

## Conclusions

Individuals with IBD reported various individual-, social-, and societal-level factors influencing sleep health. Our findings emphasize the significance of considering not only disease activity but also mental health, family, work, and the natural environment when addressing sleep health in this population. In short, this study highlights the need for tailored sleep interventions that consider these unique multilevel factors experienced by those with IBD and their potential impact on intervention engagement. Healthcare providers in IBD clinics should assess and prioritize sleep health using an interdisciplinary approach to holistically address the needs of those with IBD. Future research should also consider the impact of SDoH on sleep health and advocate for policy changes aimed at addressing these broader societal-level factors.

## Data Availability

Data not publicly available.
